# Dietary Habits, TCM Constitutions, and Obesity: Investigating the Protective Effects of Vegetarian Dietary Patterns in Taiwan

**DOI:** 10.3390/healthcare13141641

**Published:** 2025-07-08

**Authors:** Po-Yu Huang, Chien-Hsiun Chen, Yen-Feng Chiu, Hong-Chun Lin, Ching-Mao Chang

**Affiliations:** 1 Taipei City Hospital, Linsen Chinese Medicine & Kunming Branch, Taipei 104, Taiwan; dr.huangboyu@gmail.com; 2 School of Chinese Medicine, College of Medicine, National Yang Ming Chiao Tung University, Taipei 112, Taiwan; 3 Institute of Biomedical Science, Academia Sinica, Taipei 115, Taiwan; chchen@ibms.sinica.edu.tw; 4 Institute of Population Health Sciences, National Health Research Institutes, Miaoli County 350, Taiwan; yfchiu@nhri.edu.tw; 5 Department of Chinese Medicine, Taoyuan Armed Forces General Hospital, Taoyuan 325, Taiwan; linhcdr@gmail.com; 6 Institute of Traditional Medicine, National Yang Ming Chiao Tung University, Taipei 112, Taiwan; 7 Autoimmune Disease and Dry Eye Syndrome Objective Measurements Lab (AIDDES Lab), National Yang Ming Chiao Tung University, Taipei 112, Taiwan; 8 Center for Traditional Medicine, Taipei Veterans General Hospital, Taipei 112, Taiwan

**Keywords:** obesity, Traditional Chinese Medicine, body constitution, Phlegm stasis

## Abstract

**Background:** Obesity is a global health challenge associated with metabolic and cardiovascular diseases. Traditional Chinese Medicine (TCM) body constitution theory offers a unique perspective on individual susceptibility to obesity; however, its integration into public health strategies remains underexplored. **Objective:** To examine the associations between vegetarian dietary patterns, TCM body constitution types (Phlegm stasis, Yang deficiency, and Yin deficiency), and overweight/obesity in a large-scale national cohort. **Methods:** Data were obtained from 3597 participants enrolled in the Taiwan Biobank. Socio-demographic variables, lifestyle behaviors (diet, smoking, physical activity), and anthropometric indicators (BMI and waist circumference) were assessed. Participants were categorized by weight status and TCM body constitution. Polytomous logistic regression models were used to evaluate associations between vegetarian dietary patterns, constitution types, and overweight/obesity, adjusting for potential confounders. **Results:** Among participants (mean age, 50.1 ± 9.4 years), 55.6% had normal BMI, 27.3% were overweight, and 17.1% were obese. Vegetarian dietary patterns were significantly associated with lower odds of Phlegm stasis (OR: 0.96; *p* < 0.001), Yang deficiency (OR: 0.97; *p* < 0.001), and Yin deficiency (OR: 0.97; *p* < 0.001), as well as with lower odds of overweight (OR: 0.72; *p* < 0.05) and obesity (OR: 0.67; *p* < 0.05). Physical activity was also associated with lower odds of all three constitution types and obesity. Phlegm stasis constitution was associated with higher odds of obesity (range of ORs: 1.18–1.58; *p* < 0.001). **Conclusions:** Vegetarian dietary patterns and regular physical activity were associated with lower odds of obesity and TCM constitution imbalances, particularly Phlegm stasis. These findings suggest a potential role for constitution-informed strategies in obesity-related public health approaches. Longitudinal studies are warranted to clarify temporal relationships and mechanisms. **Clinical Trials Registration:** ClinicalTrials.gov NCT03938207 (Study Start: 1 October 2022).

## 1. Introduction

Obesity is a major risk factor for cardiovascular diseases, metabolic syndrome, inflammatory conditions, cancer, and arthritis [1]. The economic burden of obesity is projected to reach between USD 48–66 billion in the United States and GBP 1.9–2 billion in the United Kingdom by 2030 [2]. Between 1980 and 2015, the global prevalence of obesity was estimated at 5.0% among children and 12.0% among adults, contributing to approximately 4.0 million deaths (range, 2.7–5.3 million) and 120 million disability-adjusted life years (range, 84–158 million) worldwide [3]. A 2017–2018 multi-country survey conducted in 12 European countries reported an overall adult obesity prevalence of 12.6%, with the highest rates in Romania (21.1%) and Greece (19.7%), and the lowest in Italy (7.5%) and France (8.8%); additionally, 48.1% of participants were either overweight or obese, with men more affected than women [4]. In Taiwan, from 1993 to 2001, the prevalence of a body mass index (BMI) over 27 kg/m^2^ was 15.9% for men and 13.2% for women [5]. During the same period, obesity and overweight rates were 19.2% and 30.5% for men, and 13.4% and 21.3% for women, respectively. These conditions were significantly associated with an increased risk of type 2 diabetes and cardiovascular diseases [6]. Moreover, obesity and overweight have contributed to increased healthcare costs associated with metabolic syndrome-related diseases in Taiwan [7].

The mechanisms underlying obesity are multifactorial and involve complex interactions among genetic predisposition, chronic inflammation, dietary intake, energy expenditure, and fat storage [1,8]. Key behavioral contributors include excessive caloric intake, reduced energy expenditure, and physical inactivity [9]. These processes are further regulated by intricate networks involving the endocrine, nervous, and immune systems, in conjunction with nutrient signaling, mechanotransduction, and the gut microbiota. Hormones and peptides such as ghrelin, neuropeptide Y, triiodothyronine, thyroxine, leptin, adiponectin, glucagon-like peptide-1, gastric inhibitory polypeptide, and insulin have all been implicated in appetite regulation, metabolic balance, and adiposity [1,9,10,11].

Traditional Chinese Medicine (TCM) body constitution is a classification framework derived from TCM theory that categorizes individuals based on intrinsic physical and psychological characteristics [12,13,14]. Common constitution types include Phlegm–Dampness, Yang deficiency, and Yin deficiency. Assessments are typically conducted using validated instruments, such as the Constitution in Chinese Medicine Questionnaire and the Body Constitution Questionnaire (BCQ), which have been widely applied in East Asian populations. These classifications have been associated with various chronic conditions, including cognitive impairment [15], cerebral infarction [16], metabolic syndrome [17], menopausal syndrome [18], peptic ulcers [19], dyslipidemia [20], diabetes [21,22], and other chronic diseases [23]. Emerging evidence suggests that dietary behaviors may influence these constitutional patterns. A study from Malaysia found that adherence to vegetarian dietary patterns was inversely associated with systemic inflammation and body mass index (BMI), particularly among individuals with constitutionally unbalanced states [2]. Additionally, metabolomic analyses conducted in Hong Kong revealed that individuals with different TCM constitution types exhibited distinct gut microbiota and metabolic signatures, supporting the objectivity and biological relevance of constitution-based classification [3].

Beyond its East Asian origins, TCM has been increasingly integrated into global healthcare systems. The World Health Organization recognizes TCM as a core component of traditional and complementary medicine, especially in the context of chronic disease management and prevention [24,25]. Countries such as the United States, Australia, China, and Germany have implemented institutional programs and clinical research integrating acupuncture, herbal therapy, and constitution-based diagnostics into conventional care frameworks [26,27]. This international uptake highlights the need to better understand TCM body constitution theory, both within local populations and from a translational perspective in precision and integrative medicine. Given the multifactorial nature of obesity and metabolic disorders, integrative medical approaches that combine traditional practices with modern biomedical science are gaining attention. TCM provides a systematic framework for personalized assessment and intervention, with constitution theory offering individualized diagnostic and therapeutic guidance [28]. Recent research has demonstrated the global clinical utility of constitution-based approaches, showing associations between specific TCM body constitutions and distinct metabolic or gut microbiota profiles, thus reinforcing their relevance to precision medicine principles [29,30].

Recent studies have increasingly explored the health implications of TCM body constitutions, with growing evidence supporting their associations with various clinical conditions. A large-scale Chinese study reported that lifestyle and dietary habits significantly influenced constitution status, with approximately half of the population exhibiting unbalanced constitutions. Notably, Phlegm–Dampness and Blood–Stasis types were associated with cardio-cerebrovascular disease and hyperlipidemia, while Dampness–Heat was linked to liver disease and osteoporosis [31]. A separate study in Shanghai, focusing on women of childbearing age, found that dietary preferences, particularly a dislike of vegetables, were associated with the Damp–Heat constitution [32]. Metabolomic profiling in adults with prediabetes revealed that individuals with Spleen Deficiency or Dampness–Heat syndromes had distinct alterations in lipid metabolism, including elevated glycerophospholipids, diglycerides, triglycerides, and bile acid-related metabolites. These findings support a potential link between TCM body constitutions and early metabolic dysregulation [33]. In Taiwan, researchers applied artificial intelligence methods—such as logistic regression, Bayesian networks, and decision trees—to examine the relationship between metabolic syndrome and constitution types. The Phlegm–Dampness constitution, present in over 90% of individuals at high risk for metabolic syndrome, was significantly associated with elevated cholesterol, blood glucose, and waist circumference [34]. Collectively, these studies highlight the potential of integrating traditional constitution theory with modern diagnostic tools to inform risk stratification and preventive strategies in metabolic health [35].

Recent evidence suggests that the Phlegm–Dampness constitution is associated with an increased risk of metabolic dysfunction–associated fatty liver disease, particularly among older adults, underscoring its clinical relevance in metabolic disorders [36]. Moreover, individuals with distinct TCM body constitution types have been shown to exhibit unique gut microbiota compositions and metabolic profiles, supporting the potential for constitution-based, personalized interventions [29,30]. Given the global burden of obesity and metabolic syndrome, elucidating the interplay between dietary patterns, TCM body constitutions, and metabolic health may inform more effective prevention and management strategies. These integrative approaches align with emerging models of individualized and precision medicine grounded in traditional medical frameworks [28].

Although constitution-based approaches have been predominantly studied in China and Taiwan, growing evidence from related East Asian systems supports their broader applicability. A scoping review of 198 cross-sectional studies in Traditional Chinese Medicine highlighted the continued use of constitution and pattern differentiation in observational research over the past two decades [37]. Additionally, constitutional models, such as Eight Constitution Medicine in Korea, have shown statistically significant associations between constitution types and metabolic syndrome, suggesting their potential relevance in preventive health strategies [38]. Similarly, studies based on Sasang typology have reported differences in digestive and metabolic profiles across constitution types, with certain types being associated with higher body mass index and gastrointestinal burden [39]. Collectively, these findings support the potential utility of constitution-based frameworks in advancing precision medicine for metabolic health.

Several studies have reported associations between the Phlegm–Dampness constitution and increased risk of overweight and obesity [30,40,41]. However, limited research has examined these associations in Taiwanese populations, and the role of dietary patterns in this context remains unclear. This study aims to investigate whether vegetarian dietary patterns are associated with a lower prevalence of the Phlegm–Dampness constitution and with reduced odds of overweight and obesity. We hypothesize that a vegetarian dietary pattern may be associated with more favorable metabolic profiles by attenuating constitution-related susceptibilities. To address these gaps, we analyzed data from 3597 participants in the Taiwan Biobank, categorized by body mass index (BMI) into normal weight, overweight, and obesity groups. Given the rising prevalence of obesity and its substantial health burden, this study explores the associations among TCM body constitutions, dietary patterns, and obesity in a Taiwanese cohort, aiming to inform future applications of constitution-based individualized medicine.

## 2. Methods

### 2.1. Study Design and Data Source

**Figure 1:**
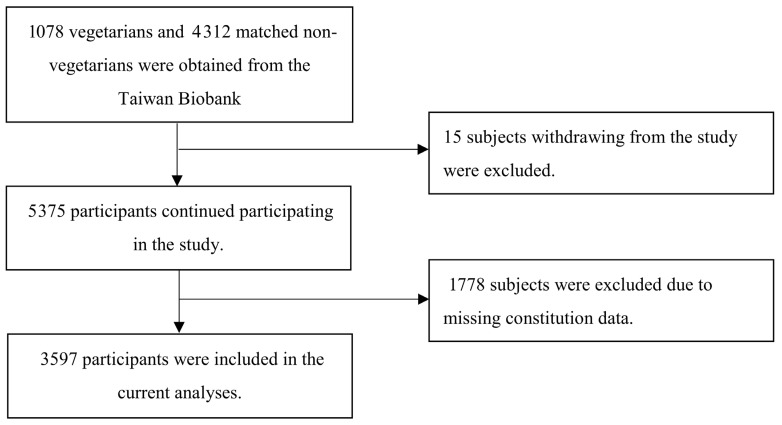
Flowchart of participant recruitment for overweight and obesity studies.

This observational, matched case-control study utilized data from the Taiwan Biobank database [42]. We identified 1073 vegetarians and matched them at a 1:4 ratio with 4302 non-vegetarians based on age and sex, forming a matched cohort. After excluding 1778 individuals lacking data on TCM body constitution, 3597 participants remained for analysis (Figure 1). Direct participant contact was not required; thus, informed consent was waived. The study protocol was approved by the Institutional Review Board of Taipei Veterans General Hospital (Approval No. 2018-04-009ACF) and was registered at ClinicalTrials.gov (NCT03938207, study start: 1 October 2022).

### 2.2. Taiwan Biobank Database

The Taiwan Biobank has systematically collected phenotypic data through standardized physical examinations and structured interviews conducted by trained personnel. These interviews capture detailed information on lifestyle behaviors, dietary patterns, environmental exposures, family medical history, and Traditional Chinese Medicine (TCM) body constitution assessments. The database also includes comprehensive demographic and clinical data, such as sex, age, marital status, residential location, educational level, employment status, alcohol and tobacco use, as well as laboratory indicators of chronic diseases including hyperlipidemia, hypertension, diabetes, and depression, along with ocular conditions such as cataracts and glaucoma.

The Body Constitution Questionnaire (BCQ) has been psychometrically validated in Taiwanese populations, demonstrating acceptable internal consistency (Cronbach’s α = 0.55–0.88) and test–retest reliability (intraclass correlation coefficient > 0.7), supporting its utility for epidemiologic studies of constitution classification [43,44,45,46,47]. The BCQ assesses physical condition over the preceding month through 44 items categorized into three constitution types: 19 items for Yang deficiency (BCQ+), 19 for Yin deficiency (BCQ−), and 16 for Stasis (BCQs). Responses are based on a five-point Likert scale and scored from 0 to 100.

The Taiwan Biobank was selected for this study due to its large, population-based cohort of over 200,000 community-dwelling adults aged 30 to 70 years. It provides standardized data across biometric, behavioral, and biochemical domains, along with validated TCM constitution assessments. Importantly, the availability of dietary habit information enables analysis of vegetarian dietary patterns in relation to metabolic outcomes and body constitution types. These features make the Taiwan Biobank an ideal platform for investigating associations between constitution, diet, and obesity in a real-world, population-relevant context.

### 2.3. Study Population

Subjects were classified as normal weight (BMI < 24 kg/m^2^), overweight (24 ≤ BMI < 27 kg/m^2^), or obesity (BMI ≥ 27 kg/m^2^) [48]. Constitution types were determined by the constitution questionnaire scores by Su et al. [43,45,49]. The constitution questionnaires included three sub-questionnaires to assess Phlegm stasis [43], Yang deficiency [44,45], and Yin deficiency constitution [46,47]. Cronbach’s α ranged from 0.55 to 0.88, and most intra-class correlation coefficients were greater than 0.7. There were 19 questions in both the Yang deficiency constitution questionnaires (BCQ+) and the Yin deficiency constitution questionnaires (BCQ−). The cut-off points were 30.5 and 29.5 for BCQ+ and BCQ−, respectively [45,47]. There were 16 questions in the Stasis Body Constitution Questionnaire (BCQs), and the cut-off point of BCQs was suggested to be 26.5 [43].

Subjects were classified into three BMI categories: normal weight (BMI < 24.0 kg/m^2^), overweight (24.0 ≤ BMI < 27.0 kg/m^2^), and obesity (BMI ≥ 27.0 kg/m^2^), based on Taiwan-specific cutoffs [48]. Body constitution types were determined using the Body Constitution Questionnaire (BCQ) developed and validated by Su et al. [43,45,49]. The BCQ comprises three subscales assessing Phlegm stasis [43], Yang deficiency [44,45], and Yin deficiency [46,47] constitutions. Cronbach’s α coefficients for these scales ranged from 0.55 to 0.88, and most intraclass correlation coefficients exceeded 0.70, indicating acceptable reliability. The Yang deficiency (BCQ+) and Yin deficiency (BCQ−) subscales each include 19 items. The validated cut-off scores for classifying individuals as having Yang or Yin deficiency were 30.5 and 29.5, respectively [45,47]. The Phlegm stasis subscale (BCQs) includes 16 items, with a recommended cut-off score of 26.5 [43].

### 2.4. Inclusion and Exclusion Criteria

Participants were selected from the Taiwan Biobank database, which enrolls community-dwelling individuals aged 30–70 years across Taiwan. For the present study, we included individuals who met the following criteria: (1) availability of complete body mass index (BMI) data; (2) valid responses to the Body Constitution Questionnaire (BCQ); and (3) complete dietary pattern information, including vegetarian dietary pattern status. Only participants with complete demographic, lifestyle, and clinical data were retained for analysis.

Exclusion criteria were as follows: (1) self-reported diagnosis of cancer, stroke, or major cardiovascular disease at baseline, to minimize potential confounding from severe illness; (2) implausible dietary intake reports, as determined by extreme caloric values (<500 kcal or >5000 kcal/day); and (3) incomplete or missing data on key covariates, such as smoking status, physical activity, or educational level.

### 2.5. Dietary Score Calculation

Dietary habits were evaluated using a validated 17-item food behavior questionnaire administered by the Taiwan Biobank, assessing fat and salt intake patterns. Items 1–8 were positively scored (0–2 points) for healthier behaviors such as avoiding fatty meat, fried food, and salty condiments. Items 9–17 were reverse-coded to reflect the adoption of low-fat and low-sodium substitutes (e.g., low-fat milk, lean meat, plant-based snacks), with total scores ranging from 0 to 51—higher scores indicating healthier dietary behavior.

In addition, the biobank survey captured habitual tea and coffee consumption, frequency of eating out, and late-night snacking patterns. The vegetarian dietary pattern was assessed through a structured question set (D20–D20b), including current status, subtype (e.g., vegan, lacto-vegetarian, ovo-vegetarian, lacto-ovo vegetarian), and duration of adherence. Individuals reporting a current vegetarian dietary pattern for more than 6 months were classified as vegetarians for this analysis.

This multidimensional dietary profiling enabled adjustment for broader dietary patterns beyond fat/salt intake alone, including supplement use and external food behaviors (e.g., D21–D24). The dietary scoring and vegetarian classification frameworks were based on previously validated Taiwanese studies.

### 2.6. Statistical Analysis

Socio-demographic characteristics (including age, sex, education, and employment), lifestyle factors (vegetarian dietary patterns, current alcohol consumption, current smoking, and exercise habits), and obesity-related indicators (BMI and waist circumference) were included in the analyses. These variables were compared across TCM body constitution types and BMI categories using the Kruskal–Wallis test for continuous variables and the chi-square test for categorical variables. In the logistic regression models evaluating TCM body constitution subtypes (dependent variables: Yin deficiency, Yang deficiency, and Phlegm stasis), the independent variables included vegetarian dietary patterns, age, sex, education, employment, and lifestyle factors (current drinking, current smoking, and exercise habits). Polytomous logistic regression models were employed to assess the associations between vegetarian dietary patterns, constitution types, and overweight/obesity, adjusting for age, sex, education level, employment, and lifestyle behaviors (current drinking, current smoking, exercise habits, and/or vegetarian dietary patterns). Participants with missing data on key variables, including BMI, vegetarian status, or constitution scores, were excluded from the final analysis. Multicollinearity among predictors was assessed using variance inflation factors (VIFs); variables with VIF > 5 were excluded from the models to reduce collinearity bias. All the statistical analyses were performed using SAS 9.4.

We employed a series of progressively adjusted models to examine the associations between various predictors and BMI status, specifically comparing overweight vs. normal weight and obesity vs. normal weight categories. The methodological framework is summarized as follows:

(1) Model 1 included basic demographic covariates (age and sex) and lifestyle factors (e.g., current alcohol use, smoking, and exercise habits) to estimate their associations with the odds of being overweight or obese. This baseline model provided an initial assessment of conventional risk factors without consideration of Traditional Chinese Medicine (TCM) body constitution types.
(2) Model 2 added Phlegm stasis constitution to the covariate set to assess its independent contribution to BMI status, thereby evaluating whether individuals with this constitution type were more likely to be overweight or obese, independent of other lifestyle and demographic factors.
(3) Model 3 further incorporated dietary habits, specifically vegetarian dietary patterns, to investigate their association with BMI status and their potential role in modulating the relationship between constitution type and obesity-related outcomes.
(4) Model 4 introduced an interaction term between vegetarian dietary patterns and Phlegm stasis constitution to evaluate whether the relationship between dietary habits and BMI differed by constitutional type. This model enabled the assessment of potential effect modification, shedding light on whether dietary interventions may yield different outcomes depending on one’s TCM body constitution.
(5) Together, these models provide a stepwise framework to disentangle the complex interplay between demographic factors, constitution type, lifestyle behaviors, and dietary patterns in relation to obesity and overweight. This layered approach supports the development of constitution-informed, individualized prevention strategies targeting metabolic health.

## 3. Results

A total of 3597 participants were included in the study, comprising 2910 individuals (80.9%) in the non-vegetarian group and 687 (19.1%) in the vegetarian group. Both groups were predominantly female, with a mean age of 50.3 ± 9.4 years in the non-vegetarian group and 49.6 ± 9.2 years in the vegetarian group (Table 1). The vegetarian group had a lower mean BMI (23.5 ± 3.5) compared to the non-vegetarian group (24.0 ± 3.6). The average waist circumference across all participants was 82.6 ± 9.6 cm, with vegetarians and non-vegetarians at 82.7 ± 9.6 cm and 81.9 ± 9.4 cm, respectively. Vegetarians reported higher rates of current drinking, smoking, and regular exercise compared to non-vegetarians. The dietary score—reflecting lower intake of meat, oils, and salt—was highest among non-vegetarians (16.0 ± 3.1), followed by the total sample (15.1 ± 5.2), and lowest in the vegetarian group (14.9 ± 5.6). In terms of Traditional Chinese Medicine (TCM) body constitution, the prevalence of Phlegm stasis, Yang deficiency, and Yin deficiency was higher among vegetarians. Participants in the normal BMI group were more likely to follow a vegetarian dietary pattern than those in the overweight or obese groups. Several variables—including education level, BMI, BMI classification, Yin deficiency, vegetarian status, current alcohol use, current smoking, exercise habits, and dietary scores—were significantly associated with diabetes status, with BMI, alcohol intake, smoking, and dietary behaviors showing the strongest associations.

Table 2 presents the associations between vegetarian dietary patterns and different TCM constitution types. Older age was associated with lower odds of Phlegm stasis (OR: 0.96; *p* < 0.001), Yang deficiency (OR: 0.96; *p* < 0.001), and Yin deficiency (OR: 0.97; *p* < 0.001). Regular exercise was also associated with reduced odds of each constitution type, with ORs of 0.70, 0.76, and 0.79, respectively (*p* values ranging from <0.001 to <0.05). Vegetarian dietary patterns showed significant associations with lower odds of Phlegm stasis (OR: 0.77; *p* < 0.05) and Yang deficiency (OR: 0.72; *p* < 0.05). The female sex was associated with significantly higher odds of Phlegm stasis (OR: 2.44; *p* < 0.001), Yang deficiency (OR: 2.27; *p* < 0.001), and Yin deficiency (OR: 1.77; *p* < 0.001).

**Figure 2:**
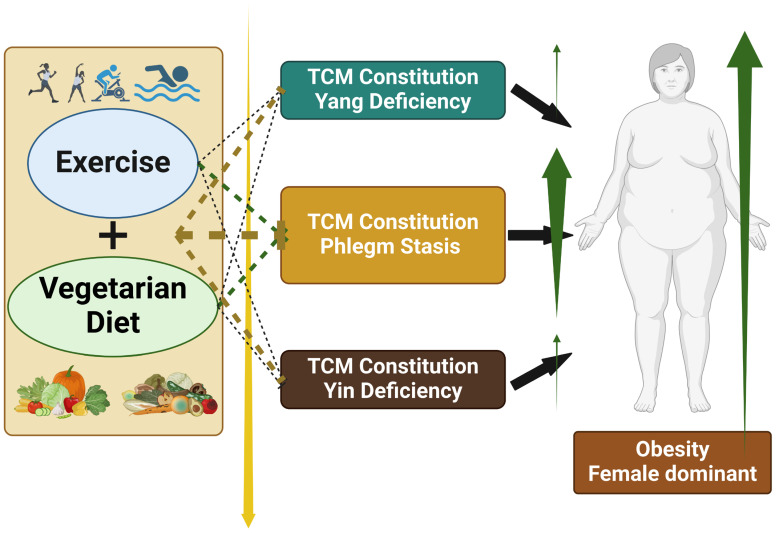
Association between vegetarian dietary patterns, Phlegm stasis constitution, and obesity. Vegetarian dietary patterns are associated with lower odds of a Phlegm stasis constitution, which, in turn, is associated with increased odds of obesity. This figure illustrates the potential pathway by which dietary habits may influence body constitution types in Traditional Chinese Medicine and how these constitution types are associated with obesity risk. The findings suggest a possible protective role of vegetarian dietary patterns against obesity through their association with body constitution modulation.

In the analysis of the association between Phlegm stasis constitution and being overweight (Table 3), age was consistently associated with higher odds (OR: 1.03) across Models 1 to 4 (*p* < 0.001). The female sex and a higher education level (college level or above) were associated with lower odds of overweight in Models 1, 2, and 3. Vegetarian dietary patterns were associated with reduced odds of overweight, with ORs of 0.67, 0.68, and 0.67 in Models 1, 3, and 4, respectively (*p* < 0.001 for all). Phlegm stasis constitution was associated with higher odds in Models 2, 3, and 4 (ORs, 1.21, 1.19, and 1.18; *p* < 0.001 for all). In the analysis of obesity, the female sex, a higher level of education (high school or above), and regular exercise were associated with significantly lower odds across all models. Vegetarian dietary patterns showed a protective association, with ORs of 0.72, 0.73, and 0.67 in Models 1, 3, and 4, respectively (*p* < 0.05 for all). Phlegm stasis constitution was associated with increased odds of obesity, with ORs of 1.58 and 1.56 in Models 2 and 3 (*p* < 0.001), and 1.44 in Model 4 (*p* < 0.05). Figure 2 illustrates the association between dietary pattern, TCM constitution, and obesity risk.

In the analysis of the association between Phlegm stasis constitution and BMI status among vegetarians and non-vegetarians (Table 4), age showed an increased odds ratio of 1.03, 1.04, and 1.02 for overweight in the entire group, for overweight in non-vegetarians, and obesity in non-vegetarians respectively, all statistically significant. Conversely, a lower odds ratio of 0.97 was observed for obesity among vegetarians. High school education was significantly associated with lower odds of obesity across all groups, including both vegetarian and non-vegetarian subgroups. Similarly, females and those with college or higher education had significantly lower odds across all groups. Exercise and dietary scores were associated with lower odds of obesity across all participants and in non-vegetarians specifically, while dietary scores also showed lower odds of overweight among non-vegetarians. However, the odds of Phlegm stasis constitution were higher at 1.50, 1.40, and 2.22 for overweight individuals across all groups, overweight non-vegetarians, and obese non-vegetarians, respectively, indicating a significant association.

## 4. Discussion

This study represents the first nationwide analysis in Taiwan to examine the interplay between TCM body constitutions, dietary patterns, and obesity. Using stratified regression models, we identified age, exercise, and vegetarian dietary patterns as significant predictors of both constitution subtypes and weight status. Age appeared to be protective against Yin deficiency, Yang deficiency, and Phlegm stasis constitutions; regular exercise was associated with a lower risk of both constitution imbalances and obesity. A vegetarian dietary pattern, in particular, was linked to reduced odds of Phlegm stasis constitution and excess body weight. These findings reflect broader patterns observed in metabolic health research, where constitution-specific susceptibilities may modulate inflammatory and metabolic responses. Distinct TCM body constitutions are believed to reflect underlying physiological tendencies that may influence an individual’s susceptibility to obesity and metabolic disorders. The role of dietary behavior, including a vegetarian dietary pattern, further highlights how constitution-informed strategies could inform integrative approaches to obesity prevention.

Our findings are consistent with recent studies demonstrating that the Phlegm–Dampness constitution is significantly associated with obesity and an elevated risk of metabolic disorders, including metabolic dysfunction–associated fatty liver disease [30]. Moreover, emerging evidence suggests that individuals with distinct TCM body constitutions exhibit unique gut microbiota compositions and metabolic profiles, providing potential mechanistic insights into how constitution types may influence metabolic health [29,30]. Recent reviews on the Dietary Inflammatory Index (DII) have further underscored the importance of dietary patterns in modulating systemic inflammation and metabolic risk. Diets with higher DII scores, which reflect a greater inflammatory potential, have been associated with increased risks of obesity, diabetes, and metabolic syndrome. In contrast, anti-inflammatory diets such as vegetarian dietary patterns may be associated with a reduction in these risks by lowering inflammatory responses and supporting metabolic balance [50]. These findings underscore that vegetarian dietary patterns may modulate TCM body constitution types and are associated with a lower risk of metabolic diseases among constitutionally susceptible individuals. Incorporating TCM body constitution assessment into clinical frameworks may thus support personalized and precision-based strategies for obesity prevention and metabolic health management [28,51]. Nonetheless, further large-scale, longitudinal studies are warranted to elucidate the underlying mechanisms and to refine constitution-based intervention models. While the TCM body constitution framework offers valuable insights for individualized prevention and therapeutic strategies, its integration into contemporary biomedical practice remains limited. This is primarily due to divergent theoretical paradigms and an incomplete understanding of the biological correlates of constitution types. Therefore, interdisciplinary research that combines metabolomics, gut microbiome profiling, and advanced statistical modeling is critical to validate TCM constitution concepts and promote their broader application in evidence-based, international clinical contexts [28,36]. Furthermore, given the cross-sectional and observational design of this study, causal relationships between body constitution, vegetarian dietary pattern, and obesity cannot be inferred. Future longitudinal and interventional studies are warranted to clarify the temporal and mechanistic pathways underlying these associations.

A potential tendency for constitutionally biased inner imbalances may predispose individuals to suboptimal health states prior to the onset of specific diseases [52], and factors such as systemic health issues, emotional dissatisfaction, and overweight could contribute to unbalanced TCM body constitutions [53], with the Phlegm–Dampness type linked to obesity [23]. TCM body constitutions of Yin deficiency, Yang deficiency, and Phlegm stasis have been found to significantly lower health-related quality of life scores [22], and internal constitutional disharmony could lead to metabolic syndrome [17]. A recent prospective study in Taiwanese patients with type 2 diabetes further showed that individuals with Yin deficiency had a significantly higher risk of all-cause mortality [54], highlighting the long-term health relevance of constitution-based profiling in TCM. In our study, aging, regular exercise, and a vegetarian dietary pattern were associated with a lower likelihood of having Yin deficiency, Yang deficiency, or Phlegm stasis constitutions, suggesting that these modifiable lifestyle factors may help prevent constitutionally unbalanced states. Furthermore, females were more likely to exhibit unbalanced constitutions compared to males, but were less likely to be overweight, regardless of constitution type.

Exercise training, including both aerobic and resistance exercises, has been shown to be effective in reducing chronic inflammation among individuals with obesity who have elevated levels of inflammatory biomarkers [55]. Our findings indicate that physical activity is associated not only with a lower likelihood of developing TCM body constitutions such as Yin deficiency, Yang deficiency, and Phlegm stasis, but also with a reduced risk of obesity. In addition to its anti-inflammatory effects, regular physical activity has been linked to improvements in TCM body constitution patterns. A population-based study involving 4497 older adults from the Taiwan Biobank demonstrated that individuals who engaged in consistent physical activity had significantly lower odds of exhibiting Yin deficiency, Yang deficiency, and Phlegm stasis constitutions compared to those who were physically inactive [56]. These findings support the view that exercise may serve as a modifiable behavioral factor that helps prevent imbalanced constitutional states and promotes both physical and psychological health.

A high-fat diet contributes to overweight and obesity by stimulating adipose tissue to secrete adipokines such as leptin, adiponectin, resistin, and visfatin. These adipokines subsequently induce pro-inflammatory cytokines, including tumor necrosis factor-α and interleukin-6, thereby promoting chronic low-grade inflammation [57]. A higher prevalence of obesity is commonly observed in low- and middle-income countries [58], and previous research has established associations between lower educational attainment and increased risk of overweight and obesity [59]. In our analysis, individuals with high school or college education exhibited a lower risk of obesity, suggesting that higher educational levels may facilitate healthier dietary choices and lifestyle behaviors, which in turn contribute to obesity prevention [60]. Furthermore, given the observed association between vegetarian dietary patterns and Phlegm stasis constitution, Model 3 included adjustments for Phlegm stasis to more accurately assess the independent effects of a vegetarian dietary pattern. Importantly, while smoking was adjusted for as a covariate in our regression models, it remains a potential confounder. Previous studies have shown that smokers tend to have lower BMI due to appetite suppression and increased metabolic rate [61]. Thus, the inverse association between vegetarian dietary pattern and overweight/obesity observed in our analysis may be partially influenced by residual confounding related to smoking habits, particularly if vegetarians in this cohort had differing smoking prevalence compared to non-vegetarians. Further stratified analyses or interaction models in future studies are warranted to clarify these relationships.

The analysis presented in Table 4 reveals a significant association between Phlegm stasis constitution and an increased likelihood of obesity among individuals following a vegetarian dietary pattern. Specifically, the odds ratio of 2.22 indicates that vegetarians with a Phlegm stasis constitution are more than twice as likely to be obese compared to those without this constitution. These findings challenge the common perception that adopting a vegetarian dietary pattern inherently confers protection against obesity. They also highlight the potential influence of TCM constitutional factors on metabolic outcomes. One possible explanation is that individuals with Phlegm stasis constitution may adopt a vegetarian dietary pattern as a reactive measure to deteriorating metabolic health. However, the benefits of such dietary changes may not be immediately observable. In TCM theory, Phlegm stasis is closely linked to metabolic dysfunction and weight gain, suggesting that even nutritionally mindful choices such as a vegetarian dietary pattern might be insufficient to counteract constitution-based susceptibility to obesity. The complex interplay of contributing factors, such as dietary habits, environmental exposures, genetic predispositions, and comorbid health conditions, further complicates the management of obesity. Thus, although vegetarian dietary patterns typically involve reduced intake of fat and salt, such modifications alone may not adequately address excess body weight [62]. These findings underscore the importance of incorporating constitutional health status into dietary counseling and obesity prevention strategies. Tailoring dietary recommendations for individuals who follow a vegetarian dietary pattern based on their TCM body constitution could enhance the precision and effectiveness of such interventions. Ultimately, integrating constitutional assessments into dietary planning may lead to more individualized and impactful approaches that better align with each person’s metabolic and constitutional profile [35].

The findings of this study offer practical implications for both clinical practice and public health policy. By identifying significant associations between TCM body constitutions and obesity levels, the results support the utility of constitution-based screening to stratify individuals at higher metabolic risk. For healthcare professionals, this provides a culturally relevant, personalized framework to guide dietary counseling and lifestyle interventions. For policymakers, the data underscore the potential of integrating constitution-based preventive strategies within community health programs, particularly in regions with established TCM infrastructure. Furthermore, incorporating constitution assessment tools into national health surveys or obesity prevention guidelines may enable earlier identification of vulnerable populations and more targeted resource allocation. These integrative approaches align with global trends toward personalized and precision medicine in chronic disease prevention.

This study has several limitations. First, the exclusion of 1778 participants due to missing TCM body constitution data may have introduced selection bias and potentially led to underestimation of the observed associations. Second, we were unable to differentiate among specific types of vegetarian dietary patterns (such as vegan, lacto-vegetarian, or ovo-lacto-vegetarian), which may exert differential effects on TCM body constitution and obesity risk. Third, the overall sample size was relatively modest; therefore, expanding the cohort and including additional participants in future waves of data collection will be essential to improve statistical power and validate the generalizability of our findings.

Despite the limitations noted, this study has several notable strengths. First, it utilizes data from the Taiwan Biobank, a large and well-characterized population-based cohort, allowing for robust statistical analyses and generalizability to middle-aged and older adults in Taiwan. Second, the use of a psychometrically validated BCQ ensures a reliable assessment of TCM body constitutions within an epidemiological framework. Third, the availability of comprehensive demographic, lifestyle, and biometric data enables the integration of constitution, dietary behavior, and obesity outcomes within a single analytic model. These methodological advantages enhance the interpretability and public health relevance of our findings and support the potential utility of TCM body constitution-based profiling in preventive health strategies.

## 5. Conclusions

This study offers important insights into the associations between dietary patterns, TCM body constitution, and obesity within a large Taiwanese cohort. Nevertheless, several limitations should be acknowledged. First, the cross-sectional design limits the ability to infer causal relationships. Second, the classification of constitution types was based solely on self-reported questionnaires, without validation using objective biomarkers. Third, the generalizability of these findings may be limited to Taiwanese populations due to cultural and dietary differences. To address these limitations, future research should employ longitudinal study designs, integrate microbiome or metabolomic profiling to validate constitution classifications, and conduct cross-cultural investigations in other East Asian and Western populations. Such efforts may enhance the scientific rigor of constitution-based approaches and clarify their relevance in the global context of metabolic health management.

## Figures and Tables

**Table 1:** Sociodemographic, lifestyle, and constitution-related variables by Body Mass Index status.

Characteristic	All	Non-Vegetarian	Vegetarian
N	3597	2910 (80.9)	687 (19.1)
Age	50.1 ± 9.4	50.3 ± 9.4	49.6 ± 9.2
Female	2568 (71.4)	2082 (71.5)	486 (70.7)
Education *			
Middle school or less	549 (15.3)	422 (14.5)	127 (18.5)
High school	1298 (36.1)	1074 (36.9)	224 (32.6)
College or higher	1750 (48.7)	1414 (48.6)	336 (48.9)
Employment	2257 (63.2)	1805 (62.5)	452 (66.1)
BMI **	23.9 ± 3.5	24.0 ± 3.6	23.5 ± 3.5
BMI status *			
Normal	2001 (55.6)	1576 (54.2)	425 (61.9)
Overweight	981 (27.3)	824 (28.3)	157 (22.9)
Obesity	615 (17.1)	510 (17.5)	105 (15.3)
Waist circumference (cm)	82.6 ± 9.6	82.7 ± 9.6	81.9 ± 9.4
Phlegm stasis	570 (15.8)	476 (16.4)	94 (13.7)
Yang deficiency	819 (22.8)	678 (23.3)	141 (20.5)
Yin deficiency *	838 (23.3)	706 (24.3)	132 (19.2)
Current drinking **	155 (4.3)	150 (5.2)	5 (0.7)
Current smoking **	230 (6.4)	214 (7.4)	16 (2.3)
Exercise habits *	1659 (46.1)	1377 (47.3)	282 (41.0)
Dietary score **	15.1 ± 5.2	14.9 ± 5.6	16.0 ± 3.1

Data are presented as either the number (percentage) or as the mean ± standard deviation. The Kruskal–Wallis test or the chi-square test was conducted to detect differences between participants with diabetes and normal glucose levels. Significantly associated with diabetes: * *p* < 0.05, ** *p* < 0.001.

**Table 2:** Associations between vegetarian dietary patterns and TCM body constitution.

Variable	Phlegm Stasis	Yang Deficiency	Yin Deficiency
Age	0.96 (0.95, 0.97) **	0.96 (0.96, 0.97) **	0.97 (0.96, 0.98) **
Female	2.44 (1.88, 3.16) **	2.27 (1.83, 2.82) **	1.77 (1.44, 2.17) **
Employment	1.04 (0.84, 1.29)	0.95 (0.79, 1.15)	0.89 (0.74, 1.07)
Drinking	0.80 (0.45, 1.41)	1.10 (0.71, 1.71)	1.13 (0.75, 1.71)
Smoking	1.09 (0.71, 1.68)	1.12 (0.78, 1.62)	1.16 (0.82, 1.65)
Exercise	0.70 (0.57, 0.85) **	0.76 (0.64, 0.90) *	0.79 (0.67, 0.93) *
Vegetarian	0.77 (0.60, 0.98) *	0.83 (0.67, 1.02)	0.72 (0.58, 0.89) *

Data are presented as the odds ratio (95% confidence interval). * *p* < 0.05 (Bold), ** *p* < 0.001 (Bold).

**Table 3:** Association between Phlegm stasis constitution and Body Mass Index status.

Predictor	Model 1		Model 2		Model 3		Model 4	
	Overweight vs. Normal	Obesity vs. Normal	Overweight vs. Normal	Obesity vs. Normal	Overweight vs. Normal	Obesity vs. Normal	Overweight vs. Normal	Obesity vs. Normal
Age	**1.03** (1.02, 1.04) **	1.00 (0.99, 1.02)	**1.03** (1.02, 1.04) **	1.01 (0.99, 1.02)	**1.03** (1.02, 1.04) **	1.01 (0.99, 1.02)	**1.03** (1.02, 1.04) **	1.01 (0.99, 1.02)
Female	**0.35** (0.29, 0.43) **	**0.39** (0.31, 0.48) **	**0.36** (0.29, 0.43) **	**0.37** (0.30, 0.47) **	**0.35** (0.29, 0.42) **	**0.37** (0.29, 0.46) **	**0.35** (0.29, 0.42) **	**0.37** (0.29, 0.46) **
Education								
Middle school or less	1.00	1.00	1.00	1.00	1.00	1.00	1.00	1.00
High school	**0.77** (0.60, 0.98) *	**0.59** (0.45, 0.78) **	0.78 (0.62, 1.00) *	**0.60** (0.45, 0.78) **	0.76 (0.60, 0.97) *	**0.58** (0.44, 0.77) **	**0.76** (0.60, 0.97) *	**0.58** (0.44, 0.77) **
College or higher	**0.58** (0.45, 0.74) **	**0.39** (0.29, 0.51) **	**0.59** (0.46, 0.76) **	**0.39** (0.29, 0.52) **	**0.58** (0.45, 0.74) **	**0.38** (0.29, 0.51) **	**0.58** (0.45, 0.74) **	**0.38** (0.29, 0.50) **
Employment	1.17 (0.97, 1.41)	1.21 (0.97, 1.51)	1.17 (0.97, 1.40)	1.20 (0.96, 1.50)	1.17 (0.97, 1.41)	1.21 (0.97, 1.51)	1.17 (0.97, 1.41)	1.21 (0.97, 1.51)
Drinking	0.97 (0.65, 1.44)	1.07 (0.69, 1.67)	1.03 (0.68, 1.54)	1.14 (0.73, 1.78)	0.97 (0.65, 1.45)	1.08 (0.69, 1.69)	0.97 (0.65, 1.44)	1.07 (0.69, 1.67)
Smoking	1.00 (0.71, 1.41)	1.30 (0.90, 1.88)	1.06 (0.75, 1.49)	1.36 (0.94, 1.96)	1.00 (0.71, 1.41)	1.30 (0.90, 1.87)	1.00 (0.71, 1.41)	1.30 (0.90, 1.87)
Exercise	0.90 (0.76, 1.06)	**0.76** (0.62, 0.93) *	0.92 (0.78, 1.08)	**0.79** (0.64, 0.96) *	0.90 (0.76, 1.07)	**0.78** (0.64, 0.95) *	0.90 (0.76, 1.07)	**0.78** (0.64, 0.95) *
Vegetarian	**0.67** (0.55, 0.83) **	**0.72** (0.56, 0.92) *			**0.68** (0.55, 0.84) **	**0.73** (0.57, 0.93) *	**0.67** (0.54, 0.84) **	**0.67** (0.51, 0.88) *
Phlegm stasis constitution			1.21 (0.96, 1.52)	**1.58** (1.23, 2.02) **	1.19 (0.95, 1.50)	**1.56** (1.22, 2.00) **	1.18 (0.92, 1.51)	**1.44** (1.10, 1.89) *
Vegetarian * Phlegm stasis constitution							1.07 (0.57, 1.99)	1.58 (0.85, 2.96)

Data are presented as the odds ratio (95% confidence interval). Model 1: Adjusts for basic demographic and lifestyle factors to assess the likelihood of being overweight or obese, without considering Traditional Chinese Medicine (TCM) constitution types. Model 2: Incorporates Phlegm stasis constitution to examine its interaction with established risk factors for obesity and overweight, providing insights for tailored interventions. Model 3: Expands the analysis to include dietary habits, specifically vegetarian dietary patterns, to understand their interaction with demographic, lifestyle, and constitutional factors affecting weight management. Model 4: Introduces an interaction term between vegetarian dietary patterns and Phlegm stasis constitution, elucidating the combined effects on overweight or obesity likelihood, highlighting targeted intervention strategies. * *p* < 0.05 (Bold), ** *p* < 0.001 (Bold).

**Table 4:** Adjusted odds ratios for body mass index status by Phlegm stasis constitution, stratified by vegetarian and non-vegetarian dietary patterns.

Predictor	All		Non-Vegetarian		Vegetarian	
	Overweight vs. Normal	Obesity vs. Normal	Overweight vs. Normal	Obesity vs. Normal	Overweight vs. Normal	Obesity vs. Normal
Age	**1.03** (1.02, 1.04) **	1.01 (0.999, 1.02)	**1.04** (1.02, 1.05) **	**1.02** (1.01, 1.03) *	1.02 (0.99, 1.04)	**0.97** (0.94, 0.998) *
Female	**0.37** (0.31, 0.45) **	**0.40** (0.32, 0.50) **	**0.33** (0.27, 0.41) **	**0.40** (0.31, 0.52) **	**0.55** (0.36, 0.85) *	**0.35** (0.21, 0.58) **
Education						
Middle school or less	1.00	1.00	1.00	1.00	1.00	1.00
High school	0.79 (0.62, 1.00)	**0.60** (0.45, 0.78) **	0.75 (0.57, 0.99) *	**0.58** (0.42, 0.78) **	0.78 (0.46, 1.34)	**0.53** (0.28, 0.998) *
College or higher	**0.60** (0.47, 0.77) **	**0.40** (0.30, 0.52) **	**0.58** (0.44, 0.77) **	**0.40** (0.29, 0.55) **	**0.57** (0.33, 0.99) *	**0.27** (0.14, 0.54) **
Employment	1.16 (0.96, 1.40)	1.20 (0.96, 1.49)	1.15 (0.93, 1.41)	**1.34** (1.05, 1.72) *	1.35 (0.86, 2.13)	0.73 (0.43, 1.24)
Drinking	1.02 (0.68, 1.52)	1.12 (0.72, 1.75)	1.01 (0.67, 1.52)	1.09 (0.69, 1.73)	-	0.37 (0.035, 3.90)
Smoking	1.02 (0.72, 1.43)	1.28 (0.89, 1.85)	0.85 (0.59, 1.22)	1.32 (0.90, 1.94)	2.75 (0.95, 7.98)	-
Exercise	0.94 (0.80, 1.11)	**0.81** (0.67, 0.99) *	0.95 (0.79, 1.15)	**0.79** (0.63, 0.99) *	0.79 (0.52, 1.18)	0.94 (0.58, 1.53)
Dietary score	0.97 (0.96, 0.99) **	**0.96** (0.94, 0.98) **	**0.97** (0.96, 0.99) *	**0.96** (0.94, 0.98) **	0.99 (0.93, 1.06)	0.99 (0.92, 1.06)
Phlegm stasis constitution	1.17 (0.93, 1.46)	**1.50** (1.17, 1.92) **	1.17 (0.92, 1.50)	**1.40** (1.06, 1.84) *	1.09 (0.61, 1.96)	**2.22** (1.23, 4.02) *

Data are presented as the odds ratio (95% confidence interval). * *p* < 0.05 (Bold), ** *p* < 0.001 (Bold).

## Data Availability

Data are contained within the article.
